# Neoadjuvant sintilimab combined with chemotherapy in resectable locally advanced non-small cell lung cancer: case series and literature review

**DOI:** 10.1186/s12957-023-03194-4

**Published:** 2023-09-26

**Authors:** Cunli Yin, Bin Hu, Xi Yang, Lingna Kou, Bo Tian, Chenghao Wang, Siru Li, Bin Liu, Jun Ge

**Affiliations:** 1 School of Medicine, University of Electronic Science and Technology of China, Chengdu, 610000 China; 2 Department Thoracic Surgery, Sichuan Cancer Hospital & Institute, Sichuan Cancer Center, Affiliated Cancer Hospital of University of Electronic Science and Technology of China, Chengdu, 610000 China; 3 School of Medical and Life Sciences, Chengdu University of Traditional Chinese Medicine, Chengdu, 610000 China; 4 Department of Medical Oncology, Sichuan Cancer Hospital and Institute, Sichuan Cancer Center, Affiliated Cancer Hospital of University of Electronic Science and Technology of China, Chengdu, 610000 China

**Keywords:** Non-small cell lung carcinoma, Neoadjuvant, Chemoimmunotherapy, PD-1(programmed cell death protein 1), Sintilimab

## Abstract

**Background:**

In recent years, neoadjuvant immunotherapy with chemotherapy has shown increasing promise for locally advanced non-small cell lung cancer (NSCLC). However, to establish its clinical efficacy and safety, it is imperative to amass more real-world clinical data. This retrospective study aims to assess the safety and effectiveness of combing sintilimab, a PD-1 inhibitor, with chemotherapy as a neoadjuvant treatment modality in patients diagnosed with potentially resectable NSCLC.

**Methods:**

We retrospectively reviewed patients with stage II-III NSCLC receiving neoadjuvant chemoimmunotherapy in Sichuan Cancer Hospital between February 2021 and February 2023. Sintilimab injection (intravenously,200 mg, iv, d1, q3w) and platinum-based chemotherapy were administered intravenously every 3 weeks, with radical lung cancer resection planned approximately 4–11 weeks after the last dose. The primary endpoint of the study was pathologic complete response (pCR). The secondary endpoints were objective response rate (ORR), and safety.

**Result:**

Thirteen patients were enrolled, they were mostly diagnosed with stage III NSCLC (IIB 15.4% IIIA 38.5%; IIIB 46.2%). Most of them had pathologically confirmed squamous cell carcinoma (69.2%).

All patients received sintilimab combined with platinum-based chemotherapy for 2 to 4 cycles. Notably, none of the patients necessitated a reduction in initial dosages or treatment postponement due to intolerable adverse events. Then, all of them underwent surgical operation. Impressively, nine patients (69.2%) achieved a pathologic complete response. The objective response rate (ORR) stood at 46.15%. Nine patients experienced neoadjuvant treatment-related adverse events (TRAEs), with only one patient (7.6%) encountering a grade 4 neoadjuvant TRAE.

**Conclusion:**

Therefore, the current study suggested that neoadjuvant sintilimab plus platinum-based chemotherapy can be a safe approach in increasing the efficiency of treatment and hopefully improving the prognosis of patients with potentially resectable locally advanced NSCLC.

## Introduction

The global cancer statistics report of 2022 revealed lung cancer’s sustained status as the leading cause of cancer-related mortality, claiming an estimated 0.6 million lives (27.2%) [1]. Notably, non-small-cell lung cancer (NSCLC) comprised 80% of all newly diagnosed cases of lung cancer. For stage III patients, the overarching therapeutic objective is curative surgical resection. However, patients with sizable or invasive tumors, as well as those with inoperable conditions, have historically been treated with neoadjuvant chemotherapy and chemoradiotherapy. Nevertheless, a meta-analysis of preoperative chemotherapy demonstrated a marginal 5% increase in the 5-year overall survival rate, rising from 40 to 45% [2]. Furthermore, a phase 3 randomized trial unveiled superior objective response, pathologic complete response (pCR), and R0 resection rates in patients who underwent chemoradiotherapy prior to surgery, as compared to those treated with chemotherapy alone. In spite of all of these, the incorporation of radiotherapy yielded no enhancements in event-free survival (the primary endpoint) or overall survival, and the pCR rate remained a modest 16% [3]. This predicament calls for novel therapeutic approaches.

At present, from ICIs (immune checkpoint inhibitors) alone to combination with chemotherapy, ICIs as neoadjuvant immunotherapy for NSCLC are evolving at a rapid pace and have acquired a certain effect [4]. The primary tumor can be used to be an antigen source, and neoadjuvant can facilitate the early development of memory T cells and induce a strong adaptive antitumor response. This mechanism was first explored by J Liu et al. in preclinical mouse models of triple-negative breast cancer [5]. Their investigation involved the inoculation of 4T1.2 tumors into BALB/c FOXP3-GFP-DTR mice (FOXP3-DTR), where all regulatory T cells (Tregs), characterized by CD4 + FOXP3 + expression, were marked with green fluorescent protein (GFP) and carried the human diphtheria toxin (DT) receptor, enabling conditional depletion via DT treatment. The findings underscored the superiority of neoadjuvant Treg depletion in eradicating metastatic disease when compared to adjuvant Treg depletion immunotherapy.

The present study aimed to describe the efficacy and safety of sintilimab plus platinum-based chemotherapy as neoadjuvant chemoimmunotherapy in patients with locally advanced NSCLC.

## Materials and methods

### Patients

The study was a retrospective study conducted at the Sichuan Cancer Hospital. The comprehensive analysis of medical records from consecutive patients who underwent therapy within the timeframe spanning February 2021 to February 2023 was undertaken. Inclusion criteria for participant selection were defined as follows: (1) verification of non-small cell lung cancer (NSCLC) at stages II–III through imaging and histological assessment prior to surgery, (2) Suitability for neoadjuvant chemoimmunotherapy (involving Sintilimab along with platinum-based chemotherapy), with subsequent capability to undergo radical lung cancer surgery post-evaluation, (3) Adherence to the Eastern Cooperative Oncology Group (ECOG) performance status criterion: ECOG ≤ 1. Conversely, the exclusion criteria were enumerated as follows: (1) NSCLC, stages II–III confirmed by imaging and histologically examination before surgery, (2) feasible neoadjuvant chemoimmunotherapy (Sintilimab plus platinum-based chemotherapy) and were able to undergo radical surgery for lung cancer after assessment, (3) ECOG score standard: ECOG ≤ 1. The exclusion criteria were as follows: (1) received chemotherapy, radiotherapy, or immunotherapy previously, (2) PD-1 inhibitors or chemotherapy intolerance, (3) complicated with other malignant tumors, (4) liver, kidney, or other organ dysfunction, (5) serious uncontrolled repeated infection or other serious uncontrolled concomitant diseases.

### Drug treatment and surgical methods

Thirteen patients were enrolled and all of them received sintilimab as neoadjuvant immunotherapy. The specific cycles and concomitant neoadjuvant chemotherapy regimen were determined by the physician in charge. Surgery was planned 4–11 weeks after the last dose. All patients received radical lung cancer resection that refers to the complete resection of tumors and regional lymph nodes in strict accordance with surgical specifications. The specific surgical method and adjuvant treatment were adopted according to the individual clinical condition of the patient.

### Study endpoints

The primary endpoint of the study was pathologic complete response (pCR). The secondary endpoints were objective response rate (ORR), and safety. ORR was evaluated according to RECIST 1.1 criteria before surgery. The safety of the neoadjuvant chemoimmunotherapy was evaluated by the National Cancer Institute Common Terminology Criteria for Adverse Events (NCI CTCAE) version 5.0 [6].

### Evaluation of efficacy

Before treatment, patients had chest computed tomography (CT), positron emission tomography-computed tomography (PET/CT), and brain magnetic resonance imaging (MRI) as baseline tumor evaluation. The radiological response of the tumor was assessed after 2 cycles of neoadjuvant and before the operation according to the Response Evaluation Criteria in Solid Tumors (RECIST), version 1.1. The indicators included complete response (CR), partial response (PR), stable disease (SD), and disease progression (PD). The pathologic responses to neoadjuvant therapy were obtained from pathological specimens after surgery. Pathological complete response (pCR) was defined as tumor regression with no residual tumor on pathology.

### Statistical analysis

All statistical analyses were performed using SPSS version 27.0 (IBM, New York, USA). All the statistical tests were two-sided with a significance level of *p* < 0.05.

## Result

### Patient characteristics

In total, 13 patients were eligible and enrolled in the present study. A comprehensive depiction of their demographic and clinical attributes is presented in Table 1. The participant composition included 12 males and 1 female, with a predominant diagnosis of stage III NSCLC (IIB 15.4% IIIA 38.5%; IIIB 46.2%). Most of them had pathologically confirmed squamous cell carcinoma (69.2%). Three patients received 2 cycles, five patients received 3 cycles, and five patients received 4 cycles. The median interval time between the last neoadjuvant treatment and operation was 37 days (IQR 28–77).

**Table 1:** Patients’ demographic and clinical characteristics

Patients no	Sex	Age year	ECOG	History of tobacco use	Histologic type	cTNM	Stage	PD-L1 (TPS %)	Neoadjuvant therapy	**Cycle**
1	M	54	0	Former	A	T4N2M0	IIIB	–	Pemetrexed + carboplatin + Sintilimab	4
2	M	58	0	Current	S	T3N1M0	IIIA	–	Abraxane + cisplatin + Sintilimab	2
3	M	50	0	Current	S	T3N2M0	IIIB	1%	Abraxane + carboplatin + Sintilimab	3
4	M	58	0	Former	S	T3N2M0	IIIB	–	Abraxane + cisplatin + Sintilimab	3
5	M	58	0	Current	A	T4N2M0	IIIB	–	Pemetrexed + cisplatin + Sintilimab	4
6	M	56	0	Former	NSCLC	T3N2M0	IIIB	–	Abraxane + carboplatin + Sintilimab	4
7	M	68	0	Former	S	T4N0M0	IIIA	2%	Taxol + carboplatin + Sintilimab	3
8	M	53	0	Former	S	T2N2M0	IIIA	8%	Abraxane + cisplatin + Sintilimab	4
9	F	59	0	Never	A	T2N2M0	IIIA	–	Abraxane + carboplatin + Sintilimab	3
10	M	68	0	Former	S	T2N1M0	IIB	< 1%	Taxol + cisplatin + Sintilimab	2
11	M	67	0	Former	S	T3N0M0	IIB	60%	Abraxane + cisplatin + Sintilimab	2
12	M	53	0	Former	S	T4N0M0	IIIA	10%	Taxol + cisplatin + Sintilimab	4
13	M	65	0	Former	S	T3N2M0	IIIB	60%	Taxol + carboplatin + Sintilimab	3

*F* female, *M* male, *ADC* adenocarcinoma, *SCC* squamous carcinoma, *cTNM* clinical TNM stage

### Efficacy and safety

**Fig. 1:**
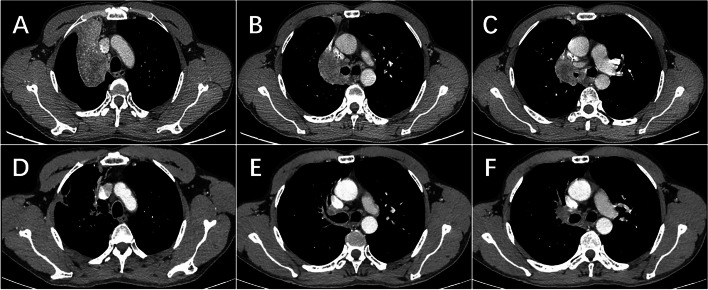
Radiological evaluation before and after neoadjuvant therapy for patient 3. **A**–**C** Initial evaluation before use of the neoadjuvant approach. **D**–**F** After 2 cycles of treatment radiological assessment

**Fig. 2:**
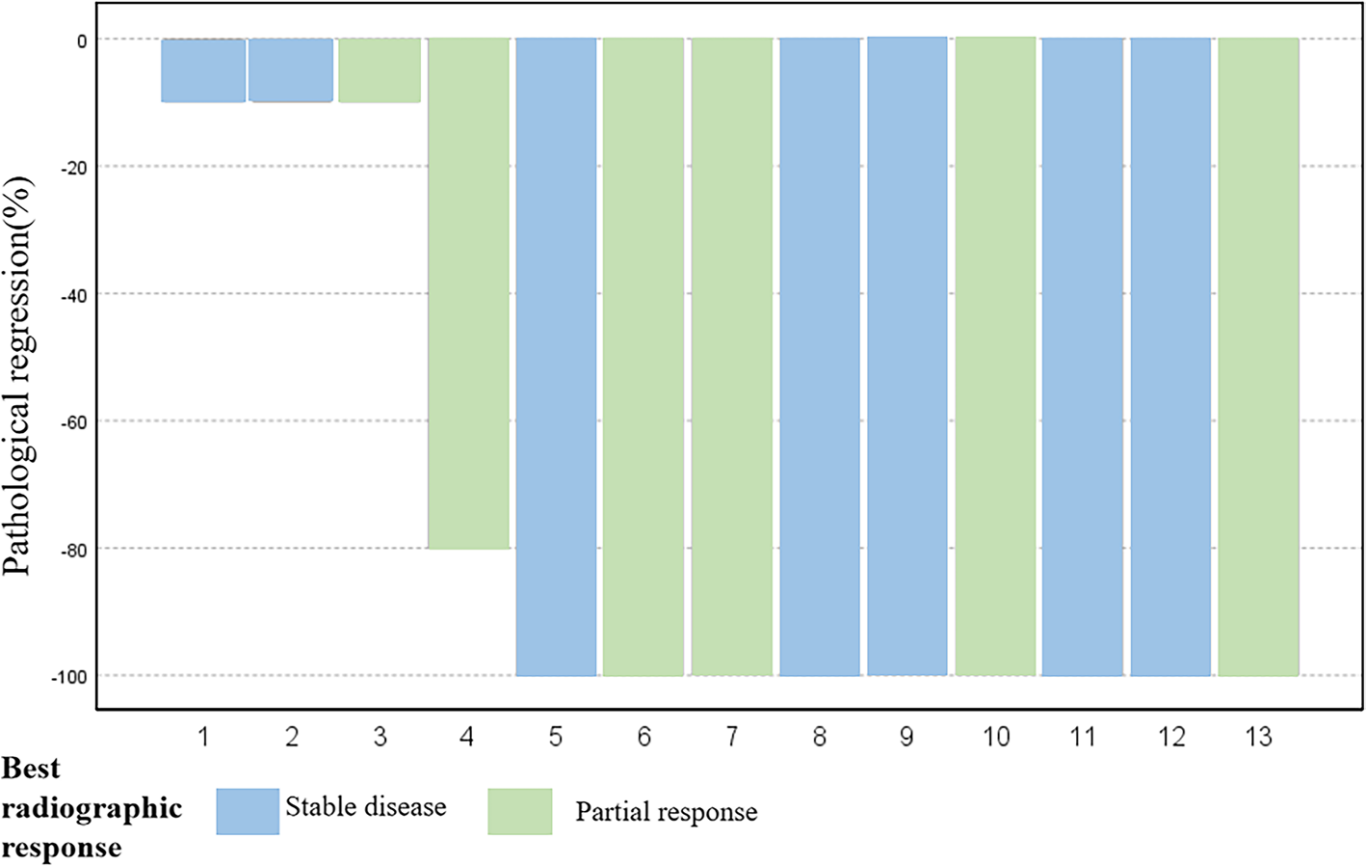
Outcomes of neoadjuvant treatment

The median follow-up time was 6 months (2–15 months), and there were no deaths among these participants. After neoadjuvant treatment, six patients achieved a partial response (PR) while seven achieved stable disease (SD) according to RECIST 1.1 criteria. Specially, the radiological assessment of patient 3 showed over 50% tumor shrinkage (Fig. 1). The objective response rate (ORR) was 46.15%. All of them underwent surgical operation (Table 2) and nine patients (69.23%) achieved pCR (Table 3 and Fig. 2 Outcomes of neoadjuvant treatment).

**Table 2:** Surgical methods

Patients no	Surgical approach	Surgical method
1	Open surgery	Bilobectomy
2	RATS	Sleeve resection
3	Open surgery	Sleeve resection
4	RATS	Lobectomy
5	Open surgery	Lobectomy
6	Open surgery	Extended resection
7	RATS	Lobectomy
8	RATS	Bilobectomy
9	VATS	Lobectomy
10	RATS	Lobectomy
11	RATS	Lobectomy
12	RATS	Lobectomy
13	VATS	Lobectomy

*RATS* robot-assisted thoracic surgery, *VATS* video-assisted thoracic surgery

**Table 3:** Outcomes of neoadjuvant treatment

Patients no	Interval time between the last neoadjuvant treatment and operation, days	The best radical response	Pathological response (pCR or no pCR)
1	28	SD	no pCR
2	30	SD	pCR
3	35	PR	pCR
4	48	PR	pCR
5	38	SD	pCR
6	37	SD	pCR
7	33	PR	pCR
8	29	SD	pCR
9	69	PR	no pCR
10	37	SD	no pCR
11	77	SD	pCR
12	37	PR	no pCR
13	46	PR	pCR

*PR* partial response, *SD* stable disease, *CR* complete response, *pCR* pathological complete response

All adverse events (AEs) were graded according to the National Cancer Institute Common Terminology Criteria for Adverse Events (NCI CTCAE) version 5.0. The recorded AEs were mainly grade 1, and only one patient experienced severe AEs (SAEs, grade ≥ 3). The majority of grade 1 AEs included decreased Hb count (62%), and increased alanine aminotransferase (46%) (Table 4). With symptomatic treatment, all patients with AEs were normalized and no AEs resulted in the discontinuation of neoadjuvant therapy.

**Table 4:** Treatment-related adverse events

Treatment-relate adverse events	Grade 1–2	Grade3	Grade4
Anemia	8 (62%)	0	0
Alanine aminotransferase Increased	6 (46%)	0	0
Constipation	1 (8%)	0	0
Rash	1 (8%)	0	0
Thrombocytopenia	3 (23%)	0	0
Leukopenia	2 (15%)	1 (8%)	0
Hypoproteinemia	3 (23%)	0	0
Hypokalemia	1 (8%)	0	0
Hyperuricemia	3 (23%)	0	0
Postoperative complications	0	0	1 (8%)

## Discussion

Numerous lines of evidence underscore the value of neoadjuvant therapy in lung cancer, as it affords the opportunity to uncover latent metastases, address comorbidities, and implement strategies such as preoperative smoking cessation and pulmonary “pre-habilitation” techniques [7]. Nevertheless, as mentioned earlier, the improvement of overall survival with neoadjuvant chemotherapy is less than satisfactory. The data of pathologic responses with neoadjuvant chemotherapy is relatively low for NSCLC (MPR < 20% and pCR ≤ 4%) [8]. The emergence of neoadjuvant immunotherapy and neoadjuvant chemoimmunotherapy has shown promise in circumventing this predicament. Notably, a meta-analysis encompassing seven studies on neoadjuvant immunotherapy indicated MPR rates spanning 18–45%, with corresponding pCR rates ranging from 4.9 to 27.3% [9]. A pivotal milestone in this field was Checkmate 159 clinical trial, which introduced neoadjuvant immunotherapy in limited-stage NSCLC patients. In this study, patients who were surgically resectable early (stage I, II, or IIIA) NSCLC received nivolumab every 2 weeks (at a dose of 3 mg per kilogram of body weight), and surgery was planned approximately 4 weeks after the first dose. This regimen induced a major pathological response in 45% (9 of 20) of resected tumors [10]. LCMC3 trial administered atezolizumab as neoadjuvant therapy, resulting in a major pathological response for 45% of the resected tumors (29 out of 143) [11]. NEOSTAR study, a phase 2, open-label, single-institution, randomized trial, evaluated Nivolumab or Nivolumab plus ipilimumab, administered intravenously. The MPR rate was 22% (5 of 23) and 38% (8 of 21) respectively [12]. And one study about neoadjuvant sintilimab was set by Li N et al., a total of 40 patients with NSCLC were enrolled. They received 2 doses of sintilimab and 37 patients underwent radical resection. Among 37 patients, 15(40.5%) patients achieved MPR, and 6 (16.2%) patients had complete pathologic response (pCR) [13].

Immunotherapy does not have a direct cytotoxic effect targeting tumor cells to reduce tumor volume, however, chemotherapy dose [14]. However, an interesting trend has emerged in recent years regarding the shifting landscape of immunotherapy trials. The average planned enrollment for immunotherapy trials has experienced a substantial decline of more than 500% over the past 7 years, plummeting from 854 participants in 2014 to 131 participants in 2020 [15]. This shift has seen combination therapies take center stage in clinical trials involving PD1/PDL1 inhibitors, gradually becoming the focus of research endeavors. This trend has also paved the way for the rise of neoadjuvant chemoimmunotherapy as a potential treatment. Several studies reported the effectiveness of neoadjuvant chemoimmunotherapy for NSCLC which seemed better than neoadjuvant immunotherapy alone, because of higher MPR and pCR rate. The most encouraging is the NADIM study [16]. Patients received nivolumab combined with paclitaxel and carboplatin for 3 cycles before surgical resection. Forty-one patients underwent surgery, the rate of MPR was 83% (34 of 41), and the rate of pCR was 63.4% (26 of 41).

**Fig. 3:**
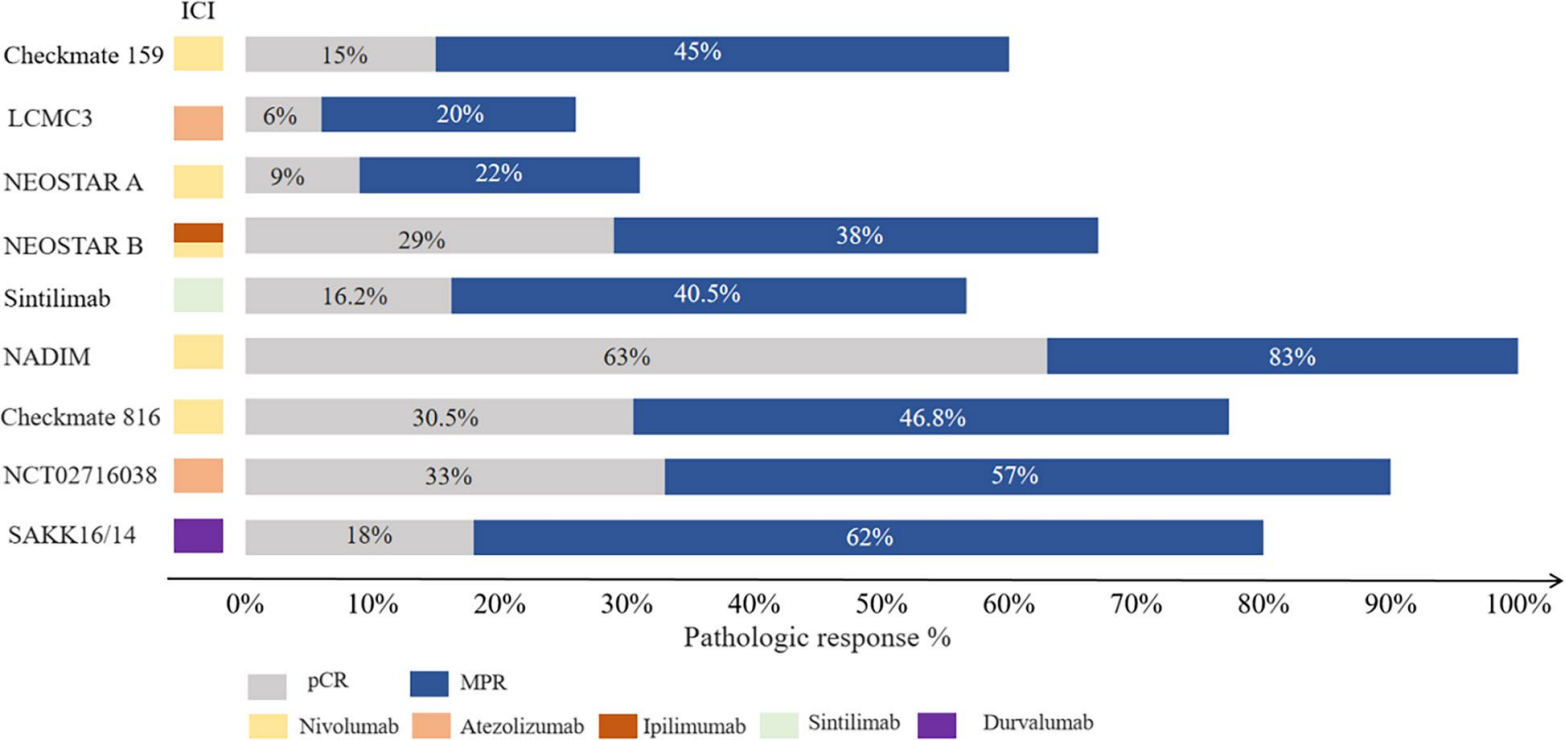
Pathologic response from clinical trials

Checkmate 816 trial, an open-label, phase III clinical study focusing on patients with stage IB–IIIA NSCLC, compared neoadjuvant nivolumab plus platinum-based chemotherapy with chemotherapy alone. In this study, the pCR rate was 24.0% versus 2.2%, and the MPR rate was 36.9% versus 8.9% [17]. NCT02716038 was an open-label, phase III trial, and patients received atezolizumab with carboplatin plus nab-paclitaxel [18]. Eleven patients were successfully operated upon, and 7/14 (50%) patients achieved MPR, including 3 patients (21%) with pCR. In SAKK16/14 trail, neoadjuvant treatment consisted of 3 cycles of cisplatin plus docetaxel followed by 2 cycles of durvalumab [19]. Finally, 55 patients underwent resection. Thirty-four patients (62%) achieved MPR, including 10 patients (18%) with pCR. We listed all studies in Fig. 3 and Table 5. Furthermore, a number of neoadjuvant chemoimmunotherapy studies were ongoing: Keynote-671 (NCT03425643), IMpower-030 (NCT03456063), and AEGEAN study (NCT03800134), and all above were randomized, double-blind, phase III clinical studies. These studies involve the administration of pembrolizumab, atezolizumab, and durvalumab in combination with platinum chemotherapy, with the objective of comparing the efficacy of neoadjuvant chemoimmunotherapy to chemotherapy alone in patients with resectable NSCLC.

**Table 5:** Characteristics and efficacy results from clinical trials assessing neoadjuvant immunotherapy

Clinical trail	Sample size	Stage	Stage III (%)	Drugs	Cycles	Surgical resection rate (%)	MPR (%)	pCR(%)
Checkmate 159	21	I–IIIA	33%	Nivolumab	2	95.2% (20/21)	45% (9/20)	15% (3/20)
LCMC3	181	IB–IIIB	49%	Atezolizumab	2	88% (159/181)	20% (29/143)^a^	6% (8/143)^a^
NEOSTAR	44	I–IIIA	22%	Nivolumab/Ipilimumab	3	82.9% (34/41)	N 22% (5/23) NI 38% (8/21)	N 9% (2/23) NI 29% (6/21)
Sintilimab (ChiCTR-OIC-17013726)	40	IB–IIIA	45.5%	Sintilimab	2	92.5% (37/40)	40.5% (15/37)	16.2% (6/37)
NADIM	41	IIIA	100%	Nivolumab + CBDCA-pacilitaxel	3	89.1% (41/46)	83% (34/41)	63% (26/41)
Checkmate 816	358	IB–IIIA	63.7%	Nivolumab + platinum-doublet chemotherapy	3	83%(149/179)	46.8%(66/14)	30.5%(43/141)
NCT02716038	30	IB–IIIA	77%	Atezolizumab + CBDCA-nab pacilitaxel	4	97%(29/30)	57% (17/30)	33% (9/30)
SAKK16/14	68	IIIA	100%	CDDP-docetaxel followed by durvalumab	3	82% (55/67)	62% (34/55)	18% (10/55)

^a^These percentages exclude patients with EGFR/ALK + . *CBDCA* carboplatin, *CDDP* cisplatin, *MPR* major pathological response (≤ 10% viable tumor cells), *pCR* pathological complete response (0% viable tumor cells)

Sintilimab can bind to PD-1, blocking the interaction of PD-1 with its ligands, and then it can help to recover the anti-tumor response of T cells. Wang J et al. conducted a surface plasmon resonance (SPR) analysis to assess the binding of sintilimab, nivolumab, and pembrolizumab to human PD-1. Their findings indicated that sintilimab exhibited the highest affinity among the three anti-PD-1 monoclonal antibodies [20]. Furthermore, an evaluation of the anti-tumor efficacy of these anti-PD-1 monoclonal antibodies in NOG mice reconstituted with human immune cells demonstrated that sintilimab treatment yielded a superior anti-tumor effect against NCI-H292 tumors when compared to nivolumab and pembrolizumab. This effect correlated with an increased number of CD8 + T cells and tumor-specific effector T cells. These results suggested that the observed higher objective response rate (ORR) and pathologic complete response (pCR) rates in our study could potentially be attributed to these mechanisms. However, it is important to acknowledge that these conclusions should be approached with caution, considering our study’s limited sample size.

In our study, patients received 2–4 cycles sintilimab plus platinum-based chemotherapy, and we performed an evaluation to update the treatment plan after every 2 cycles, which followed the expert consensus [21]. In neoSCORE trial, they found that increasing the number of cycles of neoadjuvant treatment from two to three led to a numerical improvement in MPR with good tolerability [22]. Most participants in our study received 3 cycles of treatment, while a subset underwent 4 cycles of neoadjuvant therapy after comprehensive multidisciplinary treatment (MDT) discussions, primarily driven by the requirement for adequate surgical margins. Furthermore, the consensus also recommended surgery can be performed 4–6 weeks after the last cycle of neoadjuvant immunotherapy, our study was more or less the same as it (only one participant had surgery after 11 weeks due to COVID-19). More importantly, it did encourage that the ORR was 46.15% and pCR rate was 69.23% in our study.

Our study has several limitations. It is a descriptive study conducted at a single institution which only has a few cases, and it may limit the generalizability of results because the majority of participants are male. The existing research fail to provide the relevant data of the overall survival due to the median follow-up time is short. Furthermore, our study is kind of a descriptive one, some selected participants may refuse to receive ICIs as adjuvant therapy, and it is also recommended by some other experts [15]. Meanwhile, some patients did not test for PD-L1 because the amount of samples from the preoperative biopsy is not enough to detect PD-L1, although biomarker-based selection is not essential [21]. But our study presents a promising efficacy of sintilimab plus platinum-based chemotherapy as neoadjuvant chemoimmunotherapy, and the study also makes a contribution to provide more evidence of this kind of treatment for Asian patients. All of these above-mentioned are worthy of further investigation. In future research, we will focus on studying the effects of different combination chemotherapy regimens and PD-L1 expression on patient efficacy.

## Conclusion

In conclusion, sintilimab plus platinum-based chemotherapy as neoadjuvant chemoimmunotherapy showed promising efficacy for resectable locally advanced NSCLC and adverse drug effects were acceptable. More cases, longer follow-up time, and more evaluation index are needed to confirm the long-term outcomes of this novel treatment.

## Data Availability

Data available on request from the authors.
